# Modifiable risk factors associated with the risk of developing Parkinson's disease: a critical review

**DOI:** 10.1055/s-0045-1805075

**Published:** 2025-03-19

**Authors:** Vitor Tumas, Marcelo Jhonatan Aureliano, Carlos Roberto de Melo Rieder, Artur Francisco Schumacher Schuh, Henrique Ballalai Ferraz, Vanderci Borges, Maria Carolina Soares, Dayany Leonel Boone, Carolina Candeias da Silva, Mariana Cavalcanti Costa, Delson José da Silva, Aracelle Victor do Carmo, Luana de Rezende Mikael, Bruno Lopes Santos-Lobato, Ana Lucia Zuma Rosso, Celmir de Oliveira Vilaça, Pedro Braga-Neto, André Borges Ferreira Gomes, Camila Gonçalves Monteiro Carvalho, Grace Helena Letro, Denise Hack Nicaretta, Marcus Vinicius Della Coletta, Egberto Reis Barbosa, Rubens Gisbert Cury, Francisco Eduardo Costa Cardoso, Sarah Teixeira Camargos, Ignacio Fernandez Mata

**Affiliations:** 1 Universidade de São Paulo, Faculdade de Medicina de Ribeirão Preto, Ribeirão Preto SP, Brazil.; 2 Universidade de São Paulo, Faculdade de Medicina de Ribeirão Preto, Departamento de Neurociências e Ciências do Comportamento, Ribeirão Preto SP, Brazil.; 3 Universidade Federal de Ciências da Saúde de Porto Alegre, Departamento de Neurologia, Porto Alegre RS, Brazil.; 4 Universidade Federal do Rio Grande do Sul, Departamento de Farmacologia, Porto Alegre RS, Brazil.; 5 Universidade Federal de São Paulo, Escola Paulista de Medicina, Departamento de Neurologia e Neurocirurgia, São Paulo SP, Brazil.; 6 Universidade Federal de Goiás, Hospital das Clínicas, Centro de Referência em Doença de Parkinson e Transtornos do Movimento (CerMov), Goiânia GO, Brazil.; 7 Universidade Federal do Pará, Instituto de Ciências Biológicas, Laboratório de Neuropatologia Experimental, Belém PA, Brazil.; 8 Universidade Federal do Rio de Janeiro, Hospital Universitário Clementino Fraga Filho, Serviço de Neurologia Prof. Sérgio Novis, Rio de Janeiro RJ, Brazil.; 9 Universidade Federal Fluminense, Niterói RJ, Brazil; 10 Universidade Federal do Ceará, Faculdade de Medicina, Departamento de Medicina Clínica, Fortaleza CE, Brazil.; 11 Pontifícia Universidade Católica de Campinas, Centro de Ciências da Vida, Ambulatório de Neurologia Clínica, Setor de Distúrbios do Movimento, Campinas SP, Brazil.; 12 Universidade Federal do Estado do Rio de Janeiro, Escola de Medicina e Cirurgia, Serviço de Neurologia, Rio de Janeiro RJ, Brazil.; 13 Universidade do Estado do Amazonas, Departamento de Neurologia, Manaus AM, Brazil.; 14 Universidade de São Paulo, Faculdade de Medicina, Departamento de Neurologia, São Paulo SP, Brazil.; 15 Universidade Federal de Minas Gerais, Faculdade de Medicina, Departamento de Medicina Interna, Belo Horizonte MG, Brazil.; 16 Cleveland Clinic Foundation, Lerner Research Institute, Genomic Medicine Institute, Cleveland OH, United States.

**Keywords:** Parkinson Disease, Risk Factors, Etiology, Protective Factors, Environmental Exposure

## Abstract

The etiology of Parkinson's disease (PD) is complex and multifactorial, depending on interactions involving environmental/lifestyle and genetic factors. The genetic aspects of the disease are becoming well characterized, while the environmental factors still need further investigation. In the present narrative review, we have described the most concrete evidence of associations between environmental factors and the risk of developing PD. Physical activity, healthy dietary patterns, smoking, and caffeine intake are protective factors against PD. Head trauma, consumption of milk and dairy products, and pesticide exposure were associated with a higher risk of developing PD. The associations of alcohol consumption, living in rural areas, farming, and consumption of well water with PD are still controversial. Results of several studies strongly suggest that diabetes mellitus is a risk factor for the development of PD, as well as the pre-diabetic state. Lower serum levels of uric acid were associated with an increased risk of developing PD and with worse clinical features and faster progression of symptoms. The protective effects of nonsteroidal antiinflammatory drugs use are controversial. Several other factors were potentially associated with the risk of developing PD: environmental pollutants such as organic solvents, exposure to sunlight, vitamin D deficiency, bullous pemphigoid, bipolar disorder, inflammatory bowel disease, irritable bowel syndrome, certain infections and agents, and essential tremor. Environmental factors are important risk markers for the development of PD. Understanding these risks and protective factors could lead to the implementation of risk-modifying actions for PD.

## INTRODUCTION

The etiology of Parkinson's disease (PD) is still far from being fully understood, but it is believed to be multifactorial and derived from a complex interplay involving environmental/lifestyle and genetic factors. Over the years, there has been a debate about the proportional importance of them in the etiology of the disease. At the beginning of the twentieth century, the emergence of encephalitis lethargica led some to consider that all parkinsonisms could have a viral or environmental origin. 1 The eventual familial aggregation of PD cases has always suggested a genetic origin for the disease. However, studies on twins in the 1970s and 1980s 1 showed low concordance between siblings affected by the disease, casting doubt regarding its heritability. At the same time, the description of parkinsonism by exposure to 1-methyl-4-phenyl-1,2,3,6-tetrahydropyridine (MPTP) shifted the attention to the environmental hypothesis. 1 However, no MPTP-like environmental substance was defined as the etiologic agent, and epidemiological studies at that time 1 suggested that the prevalence of the disease remained stable compared with the rates at the beginning of the century, which would not support this hypothesis.

The discovery in the last decades of the monogenic forms of PD and subsequent studies have confirmed the involvement of hereditary factors in the etiology of the disease. We now know that genetic mutations are responsible for only 10 to 15% of PD cases, while the remaining 90% are idiopathic. 2 Furthermore, genome-wide association studies have revealed that, in sporadic forms, common genetic variants with small effects may explain up to 36% of the heritability risk, suggesting that most of the risk may come from non-genetic factors. 2

Over time, several studies have accumulated evidence that environmental factors influence the risk of developing PD. Moreover, recent observations 3 have also changed concepts about epidemiology, suggesting a greater relevance of environmental factors in PD etiology. Current data from the WHO's Global Burden of Disease Study shows that PD is now the neurological condition with the fastest increase in prevalence. 3 In part, this could be due to an aging population. However, it is suspected that the incidence of the disease may also be rising due to increased exposure to the byproducts of industrialization and environmental pollution, which could be another important trigger for the “PD pandemic”. 3

Therefore, it is essential to identify the environmental/lifestyle factors associated with the risk of developing PD. One main reason is that many of them can be modifiable, enabling prevention and delay of disease onset. In the present narrative review, we will provide an overview of the environmental factors associated with the risk of developing PD. We have performed a non-systematic literature review, focusing our observations on the analysis of reviews and systematic reviews to identify the environmental factors with the most evidence of an association with PD ( Table 1 ).

**Table 1:** Relative risk of the leading environmental factors associated with the onset of Parkinson's disease

		pooled RR (95%CI)	Reference
**Habits of daily life**			
Physical activity	Highest versus lowest	0.79 (0.56–0.82)	4
	Highest versus moderate-to-high	0.71 (0.58–0.87)	4
Smoking	Current versus never	0.47 (0.40–0.56)	10
	Past versus never	0.75 (0.69–0.81)	10
Coffee consumption		0.66 (0.57–0.77)	10
Caffeine consumption		0.54 (0.34–0.84)	10
**Environmental agents**			
Exposure to pesticides		1.76 (1.56–2.04)	10
Herbicides		1.33 (1.08–1.65)	10
Insecticides		1.53 (1.12–2.08)	10
Well-water consumption		1.27 (1.08–1.49)	39
Rural living		1.52 (0.85–2.71)	39
Farming		1.28 (0.99–1.66)	39
Head trauma		1.57 (1.35–1.83)	64
**Dietary habits**			
Milk intake		1.40 (1.13–1.73),	6
**Medical aspects and comorbidities**			
Diabetes		1.31 (1.10–1.57)	10
**Biomarkers**			
High serum urate levels		0.67 (0.50–0.91)	58

Abbreviations: 95%CI, 95% confidence interval; RR, relative risk.

## PHYSICAL ACTIVITY

Physical activity is considered a protective factor against various diseases, including PD. A systematic review 4 of 8 prospective studies observed that a significantly reduced risk of developing PD was associated with the highest levels of either total physical activity (relative risk [RR]: 0.79; 95% confidence interval [95%CI]: 0.68–0.91) or moderate-to-vigorous physical activity (RR: 0.71; 95%CI: 0.58–0.87). This analysis 4 showed a dose–response relationship involving the levels of physical activity and the risk of developing PD, especially in men: vigorous physical activity provided superior protection, while light physical activity did not affect the risk. An analysis 5 excluding the practice of physical activity in the years preceding the diagnosis of PD would reduce the chance of reverse causation. Patients in the prodromal or early stages of PD were less tolerant of more intense exercise, leading to a reverse causation as a result of the disease itself. 5 Several studies have shown the benefits of physical activity on motor and non-motor symptoms in PD, and some studies have suggested that physical activity seems to slow down the progression of Parkinsonian symptoms. 5 The main limitation to these observations stems from the fact that many studies have methodological limitations and that, in most of them, the physical activity information is collected via questionnaires, which are subject to recall bias. The effects of different types of exercise are little known as well, although many suggest that aerobic exercise is the most effective. The neuroprotective mechanisms of exercise have not been fully established, but they may include effects on neuroplasticity, neuroinflammation, production of neurotrophic factors, and metabolic beneficial effects.

## SMOKING

The association of smoking with a reduced risk of developing PD has been demonstrated in several epidemiological studies. A meta-analysis 6 of 6 cohort studies calculated a pooled RR of 0.47 (95% CI: 0.40–0.56) for current versus never smoking. In five cohort studies evaluating past versus never smoking, the pooled RR was of 0.75 (95%CI: 0.69–0.81). 6 A systematic review and meta-analysis 7 of Mendelian randomization studies also concluded that genetic liability to smoking was associated with a decreased risk of PD. Other routes of use of tobacco (chewing or snorting) and exposure to environmental tobacco are also associated with a lower risk of developing PD. The number of cigarettes smoked per year and the length of time smoking have an inverse relationship with the risk of PD. 8 This observation is paradoxical to the evidence that tobacco is a significant cause of premature death from various causes and increases the risk of developing various diseases. 9 Several hypotheses have been raised to explain this inverse association. Some suspected that a reverse-causality bias could be the cause of the association, that is, that in the prodromal phase of PD, subjects would be less responsive to the effects of nicotine and would, therefore, tend to quit smoking because of the disease effects themselves. However, studies 9 addressing this issue did not support this hypothesis. Other hypothetical mechanisms could be a neuroprotective effect of certain substances in the smoke or of nicotine itself through the action of smoking against the deposition of α-synuclein on the nasal and intestinal mucous membranes or the intestinal microbiota, favoring an anti-inflammatory profile. There is evidence of nicotine's effect on activating nicotinic acetylcholine receptors that modulate dopamine release in dopaminergic terminals, which seems to stimulate dopamine release. Several studies 10 with animal models of parkinsonism demonstrate the beneficial effects of nicotine in improving motor function as well as reducing the presence of levodopa-induced dyskinesias. Nicotine also increases cytochrome P450 enzymes, increasing the endogenous inactivation of neurotoxins. A recent study 11 used a 52-week treatment with transdermal nicotine in patients with early PD and found no evidence of the drug's neuroprotective effect.

## COFFEE AND CAFFEINE

Epidemiological studies 6 have consistently confirmed an inverse association between caffeine intake and the risk of developing PD. A meta-analysis of six cohort studies 6 calculated a pooled RR of 0.66 (95%CI: 0.57–0.77) for drinking versus not drinking coffee. Because decaffeinated coffee is not protective against PD, caffeine is believed to be the essential pharmacological factor contributing to the beneficial effects of coffee on PD. 12 Other drinks with caffeine, such as teas and other caffeinated drinks, were also assessed in studies 13 14 from multiple countries, which have shown that, in these populations, different types of teas also have a protective effect, possibly independently of coffee consumption. A meta-analysis 6 of 4 cohort studies calculated a pooled RR of 0.54 (95%CI: 0.34–0.84) for the highest versus lowest caffeine consumption, and suggested that caffeine's protective effects would be weaker in women. Caffeine intake is associated with a decreased risk of PD not only when comparing consumers to non-consumers, but also for those with higher consumption, indicating a significant dose-effect relationship. 15 The time of consumption also appears to alter the effects of coffee, as demonstrated in a clinical study that found the number of years patients drank coffee was inversely correlated with a significant increase in age at PD onset. 16 Polymorphisms in the *ADORA2A* gene, which encodes the adenosine receptor, and in the *CYP1A2* gene, which encodes cytochrome P450, can modify the neuroprotective effect of caffeine. 17

The neuroprotective effect of caffeine has been postulated to be mediated by adenosine A2A receptor blockade in dopaminergic neurons, increasing dopaminergic transmission and protecting against glutamatergic excitotoxicity and oxidative stress. 18 However, it is impossible to guarantee that the effects of coffee and tea are solely due to their caffeine content, considering that they contain other substances, some of which have known antioxidant effects. Other possible explanations include reverse causality (prodromal PD reduces the tolerability or desire for caffeine) or residual confounding by another factor, such as the Parkinsonian personality or changes in reward mechanisms. Caffeine appears to have not only a protective effect against the onset of the disease, but also a symptomatic effect in PD. However, a multicenter parallel-group controlled trial 19 with PD patients showed that caffeine did not provide clinically-relevant improvement in motor manifestations of PD; therefore, the epidemiological association between caffeine and lower PD risk does not appear to be explained by its symptomatic effects.

## DIETARY INTAKE

Dietary and nutritional factors have been associated with various chronic diseases, as well as with PD. However, this association can be very complex, and it involves many factors. Longitudinal 20 21 22 23 studies have associated a healthy dietary pattern, such as the Mediterranean diet, which is characterized by a high consumption of fruits and vegetables, polyunsaturated fatty acids, plant-based proteins, and low intake of red meat and saturated fats, to a lower risk of developing PD. Healthy diets can have neuroprotective effects and prevent the development of chronic diseases, reducing oxidative stress and inflammation. A review 24 of systematic reviews found that higher consumption of carbohydrates (pooled RR: 1.24; 95%CI: 1.05–1.48) was associated with increased risk of PD. However, it is essential to highlight that the studies revealed a change in the eating behavior of patients with PD, with an excessive predilection for sugary foods, which could increase the concentrations of brain dopamine and help improve motor and non-motor symptoms. 24 On the other hand, long-term consumption of excess calories and a high glycemic load may accelerate the process of neurodegeneration, affecting insulin metabolism. 24 In practice, a healthy diet should be recommended as a form of prevention for PD. However, analyzing diet in detail is challenging and likely hinders the interpretation of potential associations with PD risk.

## HEAD TRAUMA

Trauma to the head has often been considered one of the possible causes of PD. This possible association has always been exemplified by cases of dementia pugilistica, which is a clinicopathological entity that develops in boxers who suffer repetitive cranial injuries. However, in these cases, the neuropathological findings are characterized by the marked presence of neurofibrillary clusters and amyloid plaques. Although earlier studies suggested that head trauma predisposed to dementia, later studies confirmed the association of trauma with an increased risk of PD. A meta-analysis 25 that included 19 case-control studies, 2 nested case-control studies, and 1 cohort study showed a pooled odds ratio (OR) of 1.57 (95% CI: 1.35–1.83) for the association of PD and head trauma. Reverse causation could explain these findings, but one study 26 evaluated the occurrence of head trauma during life, excluding those occurring in the ten years prior to the onset of PD, and confirmed the association between head trauma and the risk of PD. There are studies suggesting that practicing contact sports that cause repetitive mild head trauma could increase the risk of the disease, but the evidence is still disputed. The mechanisms involved are still unclear.

## MILK CONSUMPTION

The consumption of milk and dairy products has been repeatedly associated with a higher risk of developing PD. A recent meta-analysis 27 of 6 prospective studies calculated a pooled RR of 1.40 for milk intake (95%CI: 1.13–1.73), but without an apparent dose–response effect. This association has been observed in many extensive long-term longitudinal studies and in studies that considered genetic characteristics that predicted higher consumption of milk and dairy products. 28 29 On the other hand, consuming fermented milk and cheese does not seem to be associated with an increased risk of the disease. 27 It is important to note that milk consumption was not associated with the risk of developing cognitive impairment in PD. 27 Several potential mechanisms are proposed to explain this association, such as the presence of toxic substances or environmental contaminants in milk, the proinflammatory effect of milk components through their action on the intestinal microbiota, or the reduction in endogenous vitamin D and urate production induced by milk consumption. Experimental observations 30 have suggested that exosomes containing cow's milk lipids could accelerate alpha-synuclein aggregation in the central nervous system. Alternatively, reverse causation could be at play if the prodromal phase of PD induces a specific dietary preference for milk. It is interesting to note that other studies 27 have observed that milk intake reduces the risk of developing various other chronic diseases, such as hypertension, dementia, and diabetes.

## PESTICIDE EXPOSURE

Pesticides are used to control harming organisms, such as insects, molds, and weeds. Pesticides can be divided into insecticides, herbicides, and fungicides, as well as inorganic pesticides, organochlorines, organophosphates, carbamates, and pyrethroids (according to the chemical class). Pesticides can also be classified based on their use: occupational (agriculture) and household (in and around homes and gardens). In the last years, there has been a global increase in the use and commercialization of pesticides. The United States and Brazil are the countries with the most extensive use of pesticides in the world. 31 However, small countries (such as Israel, Japan, the Netherlands, the Republic of Korea) are among the top countries for pesticide use per area for cropland. 31 Evidence shows the association of pesticide chemical exposure with increased risk of PD: a meta-analysis 6 of 51 case-control and cohort studies calculated a pooled RR of 1.76 (95%CI: 1.56–2.04) for exposure versus no exposure to pesticides, and most studies were related to occupational exposure. The same review estimated a pooled RR of 1.33 (95%CI: 1.08–1.65) for herbicides and of 1.53 (95%CI: 1.12–2.08) for insecticide exposure. The association with the use of fungicides needs to be clarified.

Pesticides may be linked to the development of PD through the direct effect of chemical substances on the nervous system and gut microbiome. Different classes of pesticides present direct mechanisms of action that cause neurodegeneration, including oxidative stress, mitochondrial dysfunction, dopamine neurotoxicity, ubiquitin-proteasome system disruption, alpha-synuclein aggregation, neuroinflammation, and gut dysbiosis. 32 Regarding occupational pesticide exposure, herbicides are more used than insecticides and fungicides. Most studies 33 34 on impact of pesticides on PD have recruited participants from rural communities, usually farmworkers. In Brazil, two of the most commercialized pesticides, glyphosate and atrazine, have been associated with PD. 35 36 More data about household exposure to pesticides, which are commercialized in many forms, needs to be available. They are commonly used in high-income countries as well as in low- and middle-income countries. Still, the informal market and the incorrect use of these products in developing regions make the exposure a more significant threat. 37 Some studies 38 39 on household pesticide use have also shown that high exposure increases the risk of developing PD in the USA and Brazil. The population-attributable fraction of high household pesticide exposure, a measure of preventable new cases if a risk factor was eliminated, indicated that eliminating excessive use would reduce 10 to 20% of new PD cases. 40 Together, these results suggest that the excessive use of pesticides in occupational or household settings may increase the risk of developing PD. Considering the utility of these products for global food production and pest control in a climate-changing world, we still need to define the limits of rational and safe pesticide management. However, further studies are still required to substantiate a definite cause-effect relationship.

## ALCOHOL CONSUMPTION

Alcohol consumption has been suggested to be associated with the risk of developing PD, but these observations are highly controversial. 6 41 42 A meta-analysis 41 has demonstrated an inverse association between alcohol consumption and the risk of PD in retrospective case-control studies, but not in prospective longitudinal cohort studies. Prospective studies describe a low level of evidence for a probable decreased or increased risk of PD, and extensive and recent studies 43 44 have also shown controversial results: in some studies, PD patients presented lower alcohol consumption when compared with the controls. An explanation could be the premorbid personality of these individuals, who would be less likely to drink alcohol. The low level of dopamine in PD could also suppress alcohol addiction. In general, the studies 43 44 have a great degree of heterogeneity, which could be due to potential confounding variables, such as genetic factors. In this sense, two Mendelian randomization studies 45 46 that included genetic variants as instruments to infer causality between exposure variables and outcomes independently of confounding factors have suggested that the genetic predisposition to higher alcohol consumption is associated with a lower risk of PD. The relationship between alcohol consumption and PD remains undefined and poorly understood.

## RURAL LIVING, FARMING, AND CONSUMPTION OF WELL WATER

Canadian studies 47 first reported the risk of PD associated with living in rural areas and well-water consumption. A meta-analysis 48 considering only higher-quality studies pointed a significant association of PD risk with rural living (4 studies), with a pooled RR of 1.52 (95%CI: 0.85–2.71), well-water consumption (5 studies), with a pooled RR of 1.27 (95%CI: 1.08–1.49) and farming (8 studies), with a pooled RR of 1.28 (95%CI: 0.99–1.66). However, there is still debate over the fact that the results of the studies are contradictory, with some showing no association between living in rural areas and the risk of developing PD, and some studies even showing the opposite. 49 50 A recent systematic scoping review 51 of Australian epidemiological studies observed that, due to the small number of studies and the wide variation of prevalence rates, it was impossible to draw firm conclusions about the prevalence of PD in rural areas. On the other hand, more detailed analyses 52 53 have suggested that the frequency of PD was related to the fact that rural areas are more exposed to pesticide use by farmers and non-farmers. Otherwise, the same discussion persists for the consumption of well water. In conclusion, the association of living in rural areas, farming, and well-water consumption with PD is controversial, but only to a certain extent. These three factors are interrelated and refer to another association with environmental exposure to pesticides.

## DIABETES AND ANTIDIABETIC DRUGS

The relationship between diabetes mellitus (DM) and PD remains under debate. However, several studies strongly suggest that diabetes and prediabetes are risk factors for the development of PD. The association between type-1 DM and the risk of PD is controversial. A recent systematic review and meta-analysis 6 found that, in cohort studies, the pooled RR for the association between DM and PD was of 1.31 (95%CI: 1.10–1.57). Another recent similar study 54 concluded that prediabetes was associated with a slight increase in the RR (1.09) of developing PD (95%CI: 1.02–1.16). Studies 55 that conducted an analysis based on Mendelian randomization also observed this association. In addition, the presence of DM seems to be associated with a more severe progression of motor and non-motor symptoms. 56 Defective insulin signaling in the brain can cause changes in brain homeostasis, resulting in mitochondrial dysfunction, excessive free radical formation, amyloid aggregation, microglial activation, and cytokine production. These cellular alterations are similar to those observed in the neurodegenerative process of PD, and they could explain the aggravating effect that diabetes could have on this process.

On the other hand, antidiabetic drugs could have a beneficial and neuroprotective effect, since certain antidiabetic drugs have shown a potential neuroprotective effect in PD, especially the glucagon-like peptide-1 (GLP-1) receptor agonists and dipeptidyl peptidase-4 inhibitors (DPP-4is). 57 Preliminary studies 58 59 on exenatide and lixisenatide, both GLP-1 receptor agonists, showed potential but modest benefits in motor symptoms throughout 12 months in PD patients. Other ongoing clinical trials are evaluating the neuroprotective effect of antidiabetic drugs in PD. Long-term follow-up studies are necessary for a better understanding of the effect of antidiabetic drugs and DM PD.

## URIC ACID

Numerous studies 60 61 have linked lower serum levels of uric acid (UA) with an increased risk of developing PD, as well as with the presence of worse motor and non-motor features and faster progression of symptoms, including cognitive decline. A meta-analysis 62 summarizing the incidence of new PD cases in 6 studies (four cohorts) suggested a pooled RR of 0.67 (95%CI: 0.50–0.91) for higher serum UA and a potential dose–response relationship. A recent study 63 has shown that a cohort of prodromal PD subjects presented higher serum levels of UA than de novo PD patients, suggesting that a decrease in the levels of the UA would occur with the transition from the prodromal to the symptomatic motor phase of PD. Whether the higher serum uric acid levels observed in prodromal PD protect conversion to full-blown clinical PD will require further studies. 63 All of these findings suggest that long-term exposure to high serum urate could be linked to a delay in PD progression. 62 However, a recent systematic review 64 found no association between gout diagnosis and the risk of developing PD. Uric acid, the final enzymatic product of purine metabolism, is considered an important natural antioxidant. Low UA levels could predispose to oxidative stress.

In contrast, high levels could reduce oxidative stress, since UA acts as a free radical scavenger and iron chelator, consequently reducing the risk of PD. 65 In vitro studies 66 have also shown that UA could activate autophagy and ameliorate alpha-synuclein accumulation. A recent neuroprotection trial on early PD, 67 using oral inosine treatment dosed to increase serum UA levels, failed to show any benefit. However, future trials may explore the role of UA levels in reducing the risk of developing PD.

## NONSTEROIDAL ANTIINFLAMMATORY DRUGS

Neuroinflammation and oxidative stress are significant contributors to the pathogenesis of PD. Nonsteroidal antiinflammatory drugs (NSAIDs) could offer neuroprotection through their antiinflammatory and antioxidant properties. These drugs can prevent neuronal death by reducing the toxicity of glutamate, reactive oxygen species, and MPTP toxicity, inhibiting cyclo-oxygenase enzyme, and decreasing lipid peroxidation, mitigating neuroinflammation and oxidative damage. The association between NSAIDs and the risk of developing PD is controversial. Some studies have suggested potential neuroprotective effects of specific NSAIDs, while others have found no significant association. A prospective cohort 68 of almost 150 thousand participants and 415 incident PD cases revealed that participants reporting regular use of NSAIDs presented a lower risk of PD. However, a prospective study 69 with 6,512 participants showed no protective effect of NSAIDs against PD. Recent meta-analyses 70 71 found no significant association between the use of NSAIDs and the risk of PD. However, a recent Mendelian randomization study 72 supported a causal relationship between the use of salicylates and reduced risk of PD. These findings underscore the need for further research to clarify the role of the use of NSAIDs in the risk of developing PD and especially identify specific subgroups that might benefit from their anti-inflammatory properties. This may be due to variations in the pharmacodynamics and pharmacokinetics of individual NSAIDs, as well as genetic and environmental factors that could have contributed to the mixed results observed.

## OTHER FACTORS

Bullous pemphigoid (BP) is a chronic autoimmune vesiculobullous disease that frequently occurs in the elderly population, and a meta-analysis 73 including 8 studies showed that BP was associated with an increased risk of PD, with a pooled RR of 3.42 (95%CI: 3.01–3.87). The association involving BP, PD, and other neurologic disorders have an unknown mechanism. Bipolar disorder (BD) is a chronic disorder of mood that affects around 2% of the world population and is associated with the risk of developing PD. A meta-analysis 74 of 6 studies (2 of them prospective) reported that BD significantly increased risk of developing PD, with an OR of 3.35 (95%CI: 1.89–5.45). Studies 75 that have analyzed the prodromal phase of PD patients have also observed BD as a common problem preceding the clinical diagnosis of the disease. There are several possible explanations for this association: a shared genetic susceptibility or a neurotoxic effect of the medications used in BD. Data 6 suggest that less exposure to sunlight and vitamin D deficiency increase the risk of developing PD. Inflammatory bowel disease and irritable bowel syndrome have been associated with increased OR risk of PD, with a hazard ratio (HR) of 1.23 (95%CI:1.10–1.38) and an OR of 1.5 (95%CI: 1.29–1.75) respectively. 76 77 The mechanism of this association may be related to disturbances in the functioning of the gut-brain axis and the potentiation of inflammatory mechanisms by the gut microbiome. Exposure to certain infections and agents has been associated with a higher risk of PD, such as *Helicobacter pylori* , the hepatitis C virus, the Malassezia genus of fungi, and pneumonia infection. 6 The recent coronavirus disease 2019 (COVID-19) pandemic has alerted us to the possibility of the infection increasing the incidence of PD in the future, but this effect has not yet been proven. Some consider essential tremor a risk factor for PD, but there is no consensus, and studies have reported controversial results. 6 Inspired by the Braak's theory, which suggested that alpha-synuclein could be deposited in the digestive tract and reach the central nervous system via retrograde transport through the vagus nerve, two studies have suggested that prior truncal vagotomy or appendectomy could be associated with a lower risk of developing PD. 78

Parkinsonism has been observed in humans following exposure to solvents such as lacquer thinners, n-hexane, carbon tetrachloride, mixed solvents, and trichloroethylene (TCE). 79 Epidemiological studies 80 also suggest a potential association between occupational exposure to solvents and PD. A halogenated solvent used as a degreasing agent since the early twentieth century, TCE is released into the air from vapor release from industrial sites, or into the soil or groundwater from on-site land disposal. Trichloroethylene has been ubiquitously used worldwide, and it is now considered a significant global pollutant due to its continuous atmospheric release and persistence in subsurface environments. Extensive research suggests that TCE is a causative factor of several diseases, including cancer, fetal cardiac development, and neurotoxicity, and has also been implicated as a possible risk factor for the development of PD. A recently published study 81 showed that the risk of PD is higher in persons exposed four decades ago to TCE and other volatile organic compounds in water. Exposure to solvents and environmental pollution are suspected of increasing the risk of PD, but studies are not yet pervasive and conclusive.

The use of many drugs has been associated with the development of PD, but most of these associations are still uncertain. 6 The chronic use of calcium channel blockers, statins, and alpha-1-adrenergic receptor antagonists appears to reduce the risk of developing PD. In contrast, the use of beta-blockers and hormone replacement therapy would have the opposite effect.

## DISCUSSION

**Figure 1:**
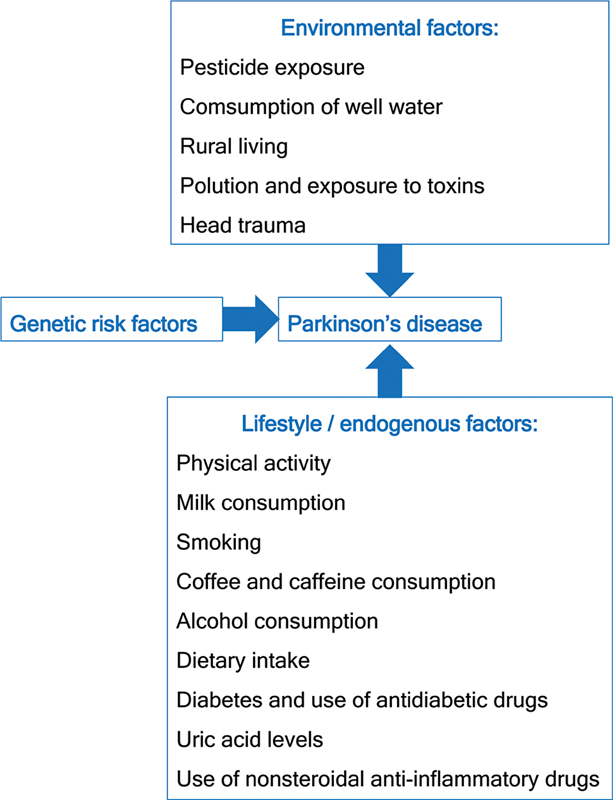
The main factors associated with the etiology of Parkinson's disease.

Environmental/lifestyle factors are currently considered very important in the etiology of PD. They probably explain 60% or more of the PD risk based on current data, alone or interacting with genetic factors ( Figure 1 ). Therefore, the Movement Disorder Society (MDS) research criteria for prodromal PD include pesticide exposure, nonuse of caffeine, nonsmoking, physical inactivity, type-II DM, and low plasma urate levels as essential risk markers for the development of PD. 82 To include these factors in the criteria, the existence of evidence based on high-quality longitudinal studies was taken into account. Despite this, many researchers still question the validity of these associations. Epidemiological studies are susceptible to various biases, mainly when based on recalling information through questionnaires. In addition, studies vary significantly in terms of how data are collected. It would be essential to have standardization so that new studies could be performed using uniform and valid instruments. The studies also need to consider the possibility that reverse causation is the reason for the association. Considering the long duration of the prodromal phase of PD, strategies need to be established to refute this hypothesis definitively for each of the factors related to the change in the risk of developing the disease. In addition, studies need to take into account the various environmental factors simultaneously to consider them as individual exposure factors in statistical analysis methods. We have focused the present review on the analysis of systematic reviews. Most participants in the studies were European, American, or Asian, and more studies on other populations are needed, such as Latin Americans. In a review 6 of systematic reviews, none of the studies were classified as having high or moderately-high quality, and most presented methodological flaws in their design or data analysis. That is why new, well-designed studies and meta-analysis are needed. One aspect that is still little explored in these studies is the perspective that genetic and environmental interactions can potentiate or minimize the effects of these factors on the subject, which is why studies of this type are highly desirable.

For the moment, discovering these risk factors could lead to implementing some risk-modifying actions for PD, especially for those subjects with a high genetic risk. It would be interesting to recommend to the population, as a way of trying to prevent the disease, the regular practice of moderate-to-intense physical activity, the consumption of caffeinated drinks and healthy water, taking caution with environmental exposure to pesticides, stimulating the adoption of the Mediterranean diet, reducing high-carbohydrate diets, and strict control of diabetes.
